# An ENU-Induced Mutation of *Nrg1* Causes Dilated Pupils and a Reduction in Muscarinic Receptors in the Sphincter Pupillae

**DOI:** 10.1371/journal.pone.0025176

**Published:** 2011-09-19

**Authors:** Bing Chen, Ke Li, Fenli Zhang, Guoqin Zhai, Wen Gong, Sujing Qiang, Zhengfeng Xue

**Affiliations:** 1 Comparative Medicine Center, Yangzhou University, Jiangsu, Yangzhou, China; 2 College of Veterinary Medicine, Yangzhou University, Jiangsu, Yangzhou, China; University of Illinois at Chicago, United States of America

## Abstract

**Background:**

N-ethyl-N-nitrosourea (ENU)-induced mutagenesis is a powerful tool for the study of gene function and the generation of human disease models. A large number of mouse mutants obtained by ENU-induced mutagenesis with a variety of phenotypes have been recovered. However, after genetic confirmation testing, only approximately 50% of the abnormal phenotypes were found to be heritable.

**Methodology/Principal Findings:**

A mouse mutant, *Dp1*, with a dilated pupil phenotype was induced with an *N*-ethyl-*N*-nitrosourea (ENU) mutagenesis strategy. Sequence analysis for *Nrg1* reveals a G>A base substitution that flanks exon E59, encoding for an EGFβ domain, in the 5′ splice donor site. The mutation affects but does not abolish the splicing of EGFβ-type *Nrg1* mRNA in *Dp1* mice and produces several different transcripts by activating other, cryptic splice sites. These types of protein isoforms are expected, and the result shows that, in the mutant, the effect is a decrease in but not an elimination of the high affinity EGFβ-type Nrg1 isoforms. This is partially compensated for by an increase in expression of the low affinity alpha forms or inactive proteins, suggesting that the mutation results in a hypomorphic allele. Interestingly, genetic model testing shows that *Dp1* is a mutation that results in a dilated pupil phenotype that is inherited with very low penetrance when heterozygous and with complete penetrance when homozygous. Pharmacological and immunohistochemical tests show a reduction of muscarinic (M) receptors in the sphincter pupillae of *Dp1* mice, which is a major cause of dilated pupils.

**Conclusions/Significance:**

This study is the first report of an *Nrg1* mutation being associated with a dilated pupil phenotype and the reduction of M receptors. This report may help in establishing more mutant mouse lines and models of human genetic disease and can be applied to other organisms. *Dp1* mice are a valuable resource for the further clarification of *Nrg1* biological function.

## Introduction

N-ethyl-N-nitrosourea (ENU) is a powerful point mutagen that can generate random mutations in the mouse genome [1]–[3]. Following an ENU-mutagenesis screen for dominant and recessive mutations, a large number of mouse mutants with a variety of phenotypes were recovered. After genetic confirmation testing, approximately 50% of the abnormal phenotypes were found to be heritable, but the rest were not (including mice that failed to breed due to illness or fertility problems and those that died before genetic confirmation testing) [4], [5]. One possible explanation for this result is that some of the abnormal phenotypes, which were not previously considered to be inheritable, are multigenic traits, rather than monogenic. Another cause could be environmental factors. However, we cannot exclude the possibility that some abnormal phenotypes, which were not previously considered to be inheritable, are heritable with a very low penetrance in the heterozygous state.

The neuregulins are a family of four genes (Nrg1–4), encoding for proteins that mediate cell-cell interactions in the brain and other organs by signaling through ErbB receptor tyrosine kinases. Neuregulin 1 (NRG1) is the most well characterized member of the family, and *Nrg1* is also a leading schizophrenia susceptibility gene [6]–[11]. Over 15 different NRG1 isoforms are produced from the single *Nrg1* gene. All isoforms contain a core EGF domain, EGFc, encoded by exon E130, but other elements of the protein are variable. There are several sources of this variation including: 1) Distinct promoter usage results in different “types” of NRG1 as defined by their 5′ exon. Currently, six types of NRG1 have been reported (types I–VI) in humans and three types (types I–III) in mice. Types I, II, IV, and V NRG1s are sometimes referred to as “Ig-NRG1”, and type III NRG1s are sometimes referred to as “CRD-NRG1”. 2) Retention of exon E68 or E59 results in the inclusion of EGFα or β variants, respectively. NRG1s with a β-type EGF sequence are predominant in the brain. 3) Most NRG1 isoforms have a transmembrane domain (TMc; exon E103) preceded by a “1” stalk (exon E24; TMc-containing NRG1s without a stalk are known as “2” isoforms); Isoforms with a “3” stalk (exon E551 in human) are truncated at that point, lack the TMc domain, and are synthesized as soluble isoforms. 4) The carboxy tail can be either the “a” or “b” form [12]–[14].

The *Dp1-Cmcyz* (Dilated pupil mutation 1, Comparative Medicine Center, Yangzhou University, hereafter *Dp1*) mutant was identified as a new ENU-induced mutant with a dilated pupil phenotype. Here, we report that the abnormal phenotype is due to a mutation in the *Nrg1* gene, which causes a reduction in muscarinic receptors in the sphincter papillae. Interestingly, the *Dp1* dilated pupil phenotype is inherited with very low penetrance in heterozygous mice and with complete penetrance in homozygous mice.

## Results

### The dilated pupil phenotype in *Dp1* mice

**Figure 1:**
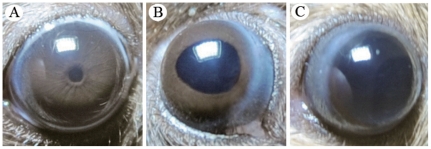
Pupil phenotype of *Dp1* mice. (A) Wild-type mouse with normal pupil. (B) *Dp1* mouse with a partially dilated pupil phenotype (heterozygous mouse). (C) *Dp1* mouse with a severely dilated pupil phenotype (homozygous mouse).

*Dp1* is an ENU-induced mutation conveying either a unilateral or bilaterally dilated pupil phenotype that can range from partial to severe (Figure 1). When illuminated, the eyes of affected *Dp1* mice have no pupillary response to light, and the sphincter pupillae fail to act.

The founder *Dp1* male mouse, which was the progeny of an ENU-treated B6 male mouse and an untreated B6 female mouse, had a unilateral dilated pupil phenotype. After mating the mutant with B6 mice, a very low percentage of the progeny (3/115) were recorded to have the unilateral dilated pupil phenotype. Interestingly, crosses among the three heterozygous progeny resulted in five out of 34 abnormal progeny, of which four had bilaterally dilated pupils. All progeny generated by crossing the above four bilaterally dilated pupil individuals with each other had a dilated pupil phenotype.

### The *Dp1* dilated pupil phenotype is caused by a mutation in the *Nrg1* gene

**Figure 2:**
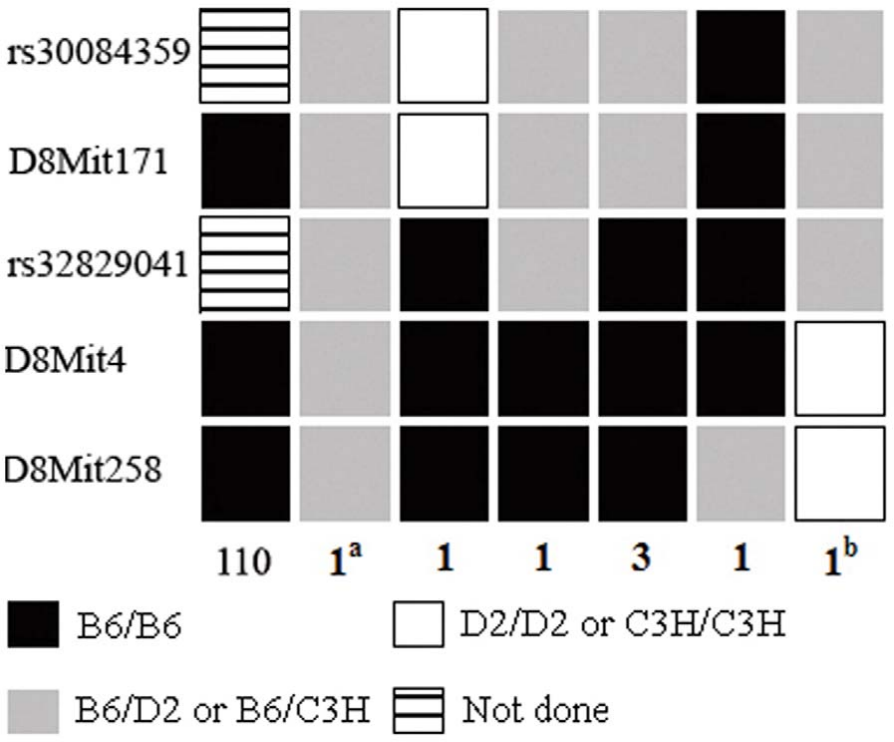
Genetic mapping places the mutant in the region between markers rs32829041 and D8Mit4. Primary markers used to refine the map position are listed on the left from the centromere (top), and the number of animals in each genotypic class is shown at the bottom. 1^a^ and 1^b^: mice with unilateral and partially dilated pupil phenotypes. The others are mice with a severe bilaterally dilated pupil phenotype.

For initial mapping, we tested genomic DNA from 25N2 samples with microsatellite markers across the whole genome. We observed no exchange of the markers *D8Mit171* and *D8Mit4* with the dilated pupil phenotype and found no significant linkages with other chromosomal loci. In order to further refine the map position, we crossbred F1 mice and reduced the critical interval to a 1.52-Mb region between the single nucleotide polymorphism (SNP) rs32829041 and *D8Mit4* using 118 F2, dilated pupil offspring (Figure 2). The region contained 7 protein coding genes (Dusp26, Rnf122, BC019943, Mak16, Fut10, 7420700N18Rik, and Nrg1), 1 miRNA gene (Mir1186) and 1 snoRNA gene (Snord13). Sequence analysis of the exons and flanking intronic sequences using DNA or mRNA of these genes revealed no apparent nucleotide changes in *Dp1* mice except for Neuregulin-1 (*Nrg1*).

**Figure 3:**
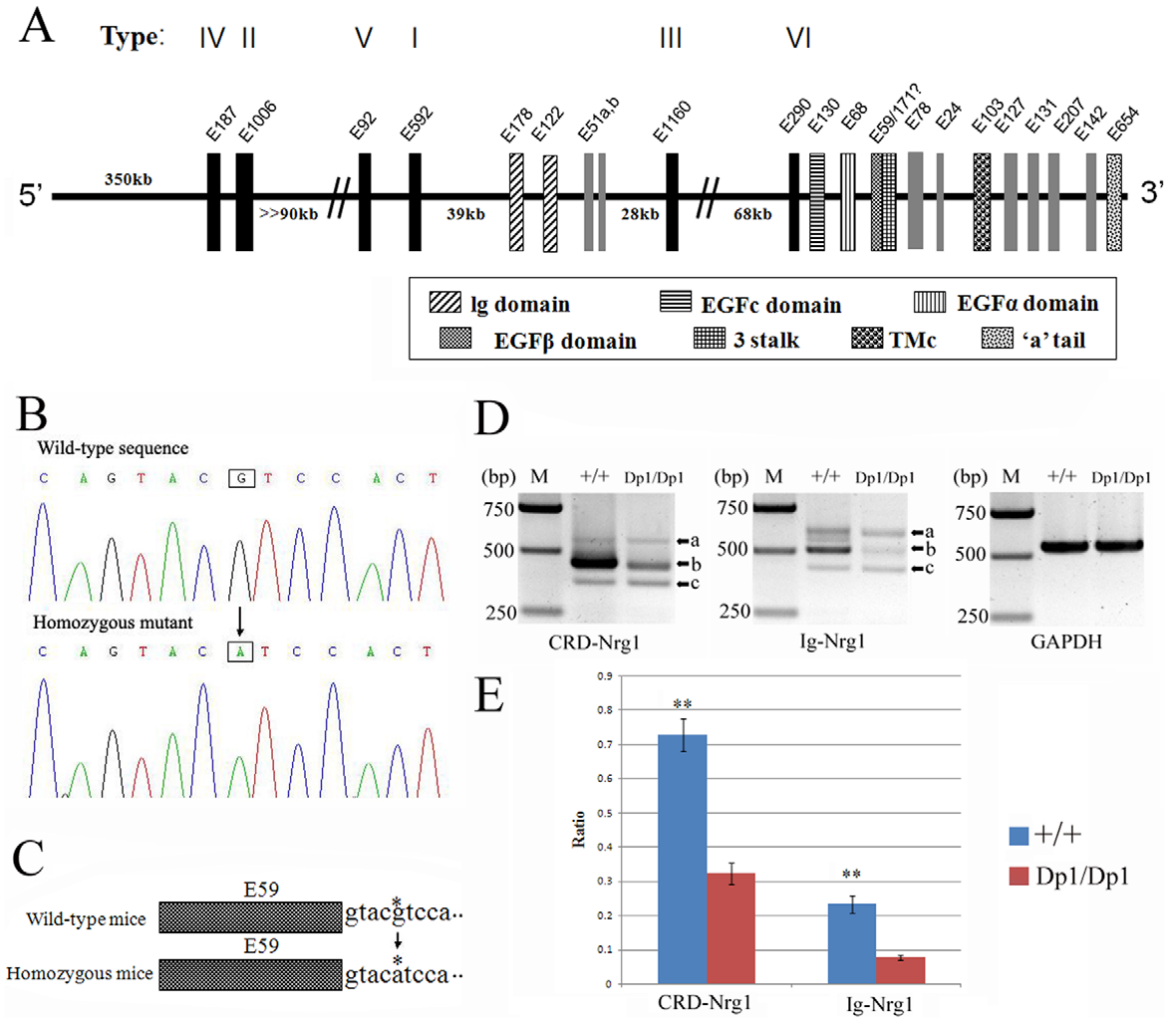
*Nrg1* gene structure and mutation identification. (A) Mouse *Nrg1* gene structure. Exons are represented as vertical bars and are numbered as per Steinthorsdottir et al. [2004], Paul et al. [2006] and Chen et al. [2008]. In the absence of an agreed or definitive exon numbering system for NRG1, exons are labeled as described by Steinthorsdottir et al. [2004] with the number denoting their length in nucleotides. The 5′ exons, which define the “types” of NRG1, are in black with the corresponding Roman numeral above (including three additional 5′ exons found in humans that encode type IV, V, and VI). In mice, exon E171 may encode isoforms with a “3” stalk (see Figure 4 and 5). (B) Sequence analysis of the *Nrg1* gene product obtained using PCR of genomic DNA discovered a G to A transition mutation in *Dp1/Dp1* homozygous mice compared to wild-type B6 mice. (C) The mutation located in the 5′ splice donor site that flanks exon E59 encoding the EGFβ domain. (D) Electrophoresis results using primers specific for CRD-Nrg1 and Ig-Nrg1 from homozygous *Dp1* and wild-type B6 mice, respectively. M, molecular weight markers. GAPDH, glyceraldehyde-3-phosphate dehydrogenase. (E) Quantitative analysis of *Nrg1* mRNA levels in band b by densitometry. Ratio: ratio of volume (intensity) of respective Nrg1 and GAPDH mRNA RT-PCR product. **p<0.01 vs. respective *Dp1/Dp1* group (n = 4).

In the *Nrg1* gene, we discovered a G>A transition mutation, which flanked exon E59, encoding for the EGFβ domain, in the 5′ splice donor site (Figure 3A–C). In order to assess the effect of the G>A substitution on EGFβ-type *Nrg1* mRNA splicing, we amplified the sequences from RNA harvested from the brains of mice homozygous for the *Nrg1* mutation and wild-type B6 mice using primers specific for *CRD-Nrg1* and *Ig-Nrg1*, respectively. For both *CRD-Nrg1* and *Ig-Nrg1* RT-PCR products, electrophoresis results revealed three bands from both *Dp1* and wild-type B6 mice (Figure 3D). The middle band (band b), corresponding to NRG1s with a β1-type EGF sequence (see below), was predominant in the brain of wild-type B6 mice. Quantitative analysis by densitometry shows that, in homozygous mice, the relative yield of band b in *CRD-Nrg1* and *Ig-Nrg1* is only 42.6% and 32.8%, respectively, of that in wild-type B6 mice (Figure 3E).

**Figure 4:**
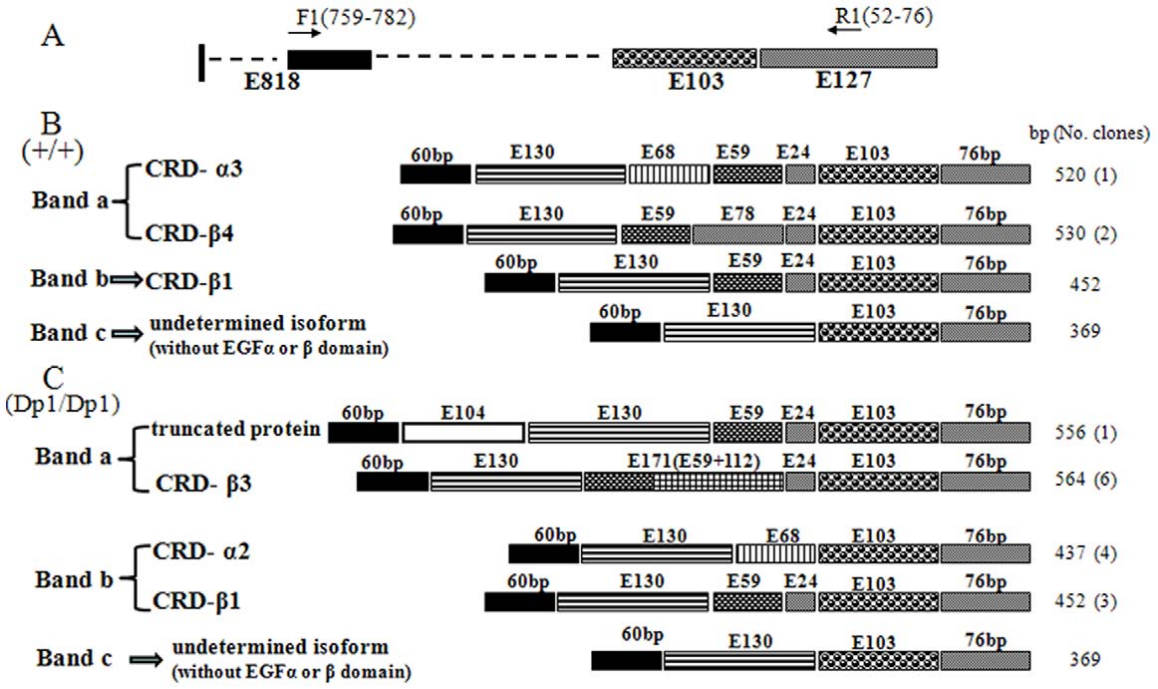
Transcripts of *CRD-Nrg1* and the expected protein isoforms in wild-type and *Dp1/Dp1* homozygous mice. (A) RT-PCR amplified region of *CRD-Nrg1* transcripts. The primers used for RT-PCR are indicated; the arrow indicates the approximate primer position within the exon. (B) Transcripts of *CRD-Nrg1* produced by wild-type mice. (C) Transcripts of *CRD-Nrg1* produced by *Dp1/Dp1* homozygous mice. In band a, one transcript contains a new exon, E104 (which is expected to produce a truncated protein without the EGFc, EGFβ, and EGFα domains), and another transcript (accounting for 6/7 of the clones) transcribes through exon E59 into the adjacent 112 bp sequence (which is expected to produce CRD-β3 type isoforms). In band b, one transcript (accounting for 4/7 of the clones) bypasses the mutated splice donor site by splicing over exon E59 (which is expected to produce CRD-α2 type isoforms).

**Figure 5:**
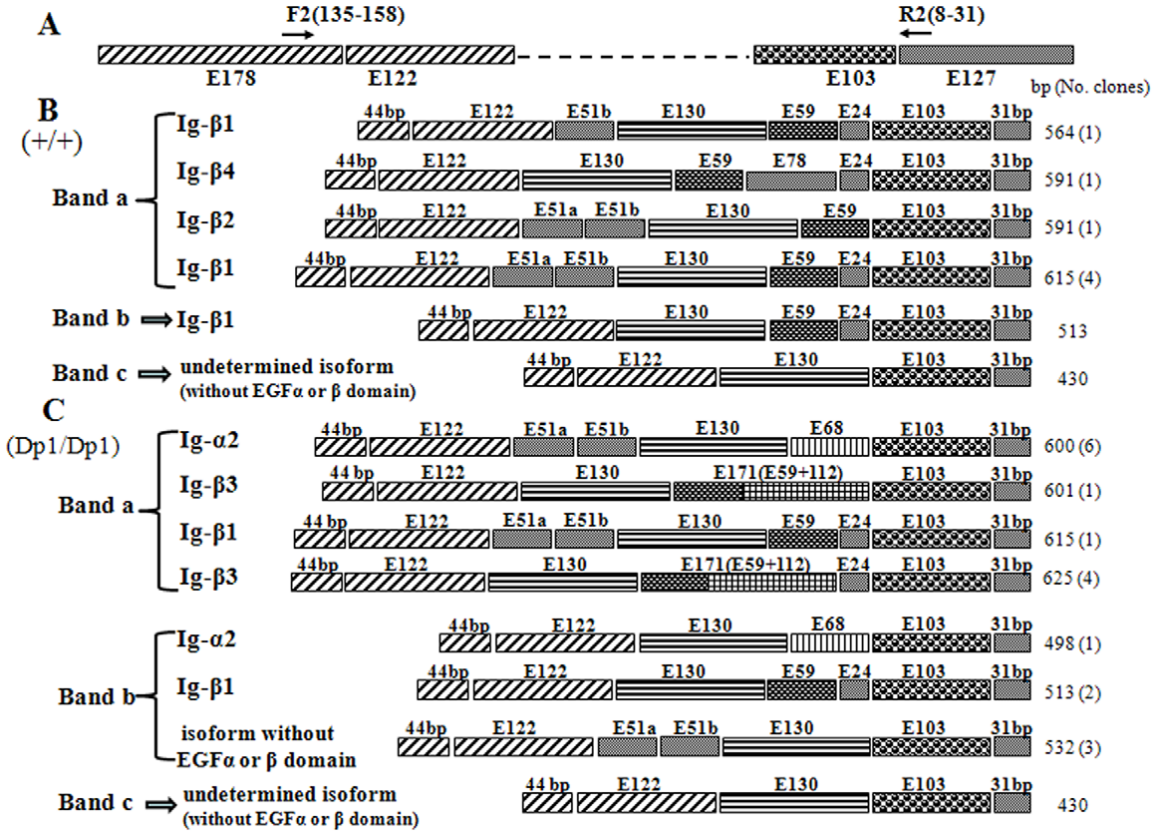
Transcripts of *Ig-Nrg1* and the expected protein isoforms produced by wild-type and *Dp1/Dp1* homozygous mice. (A) RT-PCR amplified region of *Ig-Nrg1* transcripts. The primers used for RT-PCR are indicated; the arrow indicates the approximate primer position within the exon. (B) Transcripts of *Ig-Nrg1* produced by wild-type mice. (C) Transcripts of *Ig-Nrg1* produced by *Dp1/Dp1* homozygous mice. In band a, three kinds of transcripts (accounting for 11/12 of the clones) bypass the mutated splice donor site by either splicing over exon E59, activating a cryptic splice site, or transcribing through exon E59 into the adjacent 112 bp sequence (one transcript, accounting for 6/12 of the clones, is expected to produce Ig-α2 type isoforms). In band b, two kinds of transcripts (accounting for 4/6 of the clones) bypass the mutated splice donor site by splicing over exon E59 (one transcript, accounting for 1/6 of the clones, is expected to produce Ig-α2 type isoforms and the other transcript, accounting for 3/6 of the clones, is expected to produce isoforms without the EGFβ and EGFα domains).

To determine the sequences amplified by RT-PCR analysis, individual bands generated from both wild-type and *Dp1* mice were purified and used directly as templates for nucleotide sequencing. Sequence analysis of the middle band of *CRD-Nrg1* and *Ig-Nrg1* from wild-type mice revealed wild-type sequences corresponding to CRD-β1 and Ig-β1 *Nrg1* (Figure 4B: band b; Figure 5B: band b). However, sequence analysis of the middle band of *CRD-Nrg1* and *Ig-Nrg1* in *Dp1/Dp1* mice revealed a mixed sequence (data not shown). Sequence analysis of the lowest band (band c) from both wild-type and *Dp1/Dp1* mice revealed alternative splicing transcripts that lacked exons E59 and E24 when compared to the corresponding band b sequence from wild-type mice (Figure 4B and C: band c; Figure 5B and C: band c). To our knowledge, these are new transcripts that have not previously been reported. Sequence analysis of the top band (band a) of *CRD-Nrg1* and *Ig-Nrg1* from wild-type and *Dp1/Dp1* mice revealed a mixed sequence (data not shown). We cloned the bands with mixed sequences into a T vector and then sequenced the plasmids. Sequencing of these bands revealed differences in the transcripts between *Dp1/Dp1* and wild-type mice. The majority of the transcripts found in *Dp1/Dp1* bypass the mutated splice donor site by splicing over exon E59, activating a cryptic splice site, or transcribing through exon E59 into the adjacent sequence. These types of protein isoforms were expected, and the results show that, in the mutant, there is a decrease in, but not an elimination of, EGFβ-type Nrg1 isoforms. This decrease is partially compensated for by increased expression of the alpha forms, inactive isoforms (without EGFβ, and EGFα domains), and truncated proteins (without the EGFc, EGFβ, and EGFα domains) (Figure 4B and C: bands a and b; Figure 5B and C: bands a and b).

### The dilated pupil phenotype is inherited with very low penetrance in heterozygous mice and complete penetrance in homozygous mice

To determine the inheritance pattern of the dilated pupil phenotype, genetic model testing based on genotyping for a *TaiI* restriction site polymorphism was conducted by crossbreeding unaffected *Dp1/+* mice with each other, *Dp1/Dp1* mice with unaffected *Dp1/+* mice, *Dp1/Dp1* mice with wild-type mice, and *Dp1/Dp1* mice with each other. The results show that *Dp1* is a mutation with a dilated pupil phenotype that is inherited with very low penetrance (5/111) when heterozygous and with complete penetrance when homozygous (Table 1).

**Table 1:** Genetic model testing.

Genotype. Parents	*Dp/+×Dp/+*			*Dp/Dp×Dp/+*		*Dp/Dp×+/+*	*Dp/Dp×Dp/Dp*
Total no. of offspring	n = 108			n = 11		n = 41	n = 132
Genotype of offspring	*Dp/Dp*	*Dp/+*	*+/+*	*Dp/Dp*	*Dp/+*	*Dp/+*	*Dp/Dp*
No. of affected offspring	16	3	0	4	0	2	132
Total no. of offspring of each genotype	16	63	29	4	7	41	132

In the test, all affected heterozygous offspring have a unilaterally and partially dilated pupil phenotype, but the size of the pupil varies with each individual.

Of the 152 homozygous offspring, 148 have a severe bilaterally dilated pupil phenotype, 3 show a bilateral dilated pupil phenotype with one completely and one partially dilated pupil, and 1 has a severe unilaterally dilated pupil phenotype.

Additionally, in this test, all affected *Dp1/+* mice had a unilateral and partially dilated pupil phenotype that varied in phenotypic severity, and the vast majority of the *Dp1/Dp1* mice had bilaterally dilated pupils with a severe phenotype (defined as a complete or nearly completely dilated pupil phenotype). Pan-NRG1 knockout (KO) mice (mice in which all NRG1 isoforms are unable to bind to and activate ErbB receptors due to the disruption of the EGF-like domain) and mice with all Ig-NRG1 isoforms inactivated die at E10.5 [15], [16], and mice with all CRD-NRG1 isoforms inactivated die from asphyxia at birth [17]. In *Dp1/+*×*Dp1/+* offspring, the ratio of the genotype of *Dp1/+×Dp1/+* offspring differed significantly from the expected 1∶2∶1 (homozygous∶heterozygous∶wild type) ratio (0.01<P<0.05, chi-square test), indicating an effect on viability.

### Reduction of M receptors in the sphincter pupillae causes the dilated pupil phenotype

To analyze the defect that caused the dilated pupil phenotype and cure the abnormal phenotype, we used drugs to clinically constrict or enlarge the pupils. A 1% pilocarpine solution (a nonselective muscarinic cholinergic receptor agonist) was first applied as drops in the eyes of mice with severe and partially dilated pupil phenotypes, but it did not alter pupil size in mice with either phenotype (full or partial dilation of the pupil). In contrast, administration of a 1% atropine solution, a muscarinic cholinergic receptor antagonist which can compete for AchR with Ach (we considered the drug effect to be a blocking of the AchR), resulted in the further loosening of the sphincter pupillae, which led to complete mydriasis in partially dilated pupil mice. Taken together, these results suggest that the number of muscarinic receptors might be decreased in the iris constrictor muscles of mutant mice, while the number of muscarinic receptors in partially dilated pupil mice is higher than in mice with a severely dilated pupil phenotype. The contractile extent of the sphincter corresponds to the amount of Ach and the number of AchR. Due to the decrease in AchR in partially dilated pupil mice, an excess of Ach with a limited number of AchR cannot lead to complete muscle contraction. In addition to pilocarpine, we used drops of a 1% neostigmine solution (a cholinesterase inhibitor) or both solutions together in dilated pupil mice, but both drugs failed to constrict the mutant pupils (full or partial dilation of the pupil). After intraperitoneal administration of pilocarpine or neostigmine in *Dp1* mice, severe salivation and mild lachrimation were observed, suggesting that AchRs in the glands of *Dp1* mice were not (or only slightly) affected (data not shown).

**Figure 6:**
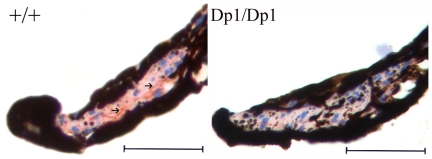
Immunohistochemistry results reveal the severe reduction of the ChRM3 receptor in the iris constrictor muscles of *Dp1* homozygotes. Representative iris sections from wild-type and homozygous mice. Staining of an anti-ChRM3 receptor antibody is shown in red and is indicated by an arrow. Scale bars: 50 µm.

M receptors comprise five distinct subtypes (M1–M5). M3 is known to play a dominant role in eliciting smooth muscles contraction, and M3−/− mice showing a partially dilated pupil phenotype have been reported [18], [19]. To further validate the reduction of M receptors in the sphincter papillae of *Dp1* mice, we performed immunohistochemistry tests using an anti-M3 antibody and found a significant reduction of M3 receptors in the sphincter pupillae of *Dp1/Dp1* mice (Figure 6).

## Discussion

We report a novel dilated pupil phenotype mouse (*Dp1*), which was obtained by ENU mutagenesis. The abnormal phenotype is caused by a base substitution flanking the EGFβ domain-encoding exon. Quantitative and sequence analysis of *Nrg1* revealed that the mutation affected, but did not abolish, the splicing of EGFβ-type *Nrg1* mRNA and produced several altered transcripts. These types of protein isoforms were expected, and the results show that, in the mutant, there was a decrease in, but not an elimination of, EGFβ-type Nrg1 isoforms. This was partially compensated for by the increased expression of alpha forms or inactive proteins. NRG1s with β-type EGF-like domain isoforms are 10–100 times more potent than NRG1s with an α-type EGF-like domain [8]. The results indicated that the mutation results in a hypomorphic allele. Although pan-NRG1, Ig-NRG1 and CRD-NRG1 KO mice have existed for many years, the relationship between *Nrg1* mutation and the dilated pupil phenotype had not been reported until now. This study is the first to report an *Nrg1* mutation associated with the dilated pupil phenotype. The *Nrg1* gene has more than 20 exons and gives rise to at least 15 different isoforms of the protein. In this paper, we also report new transcripts that had not been reported previously.

Pupil size is controlled by two different sets of involuntary muscles, the sphincter pupillae and the dilator papillae, which act in opposition to cause miosis (constriction) or mydriasis (dilation) of the pupil in response to different levels of light or during focal adjustment [20]. The sphincter muscle is innervated by the parasympathetic nervous system, which acts by releasing acetylcholine which acts on M receptors [21]. The dilator papillae are innervated by the sympathetic system, which acts by releasing noradrenaline which acts on α1-receptors [22]. Pharmacological and immunohistochemical tests showed a reduction in M receptors in the sphincter pupillae of *Dp1* mice, which is a major contributor to the dilated pupil phenotype. This is the first report of an *Nrg1* mutation being associated with the reduction of M receptors.

The role of NRG1 in mediating the nerve-dependent accumulation of AchRs in the postsynaptic membrane of nerve-muscle synapses has been previously reported. There are two kinds of AchRs: nicotinic (N) and M receptors. AchRs in the sphincter pupillae belong to the latter. As a result of the reduction of N receptors in the postsynaptic membrane, mice that are heterozygous for the deletion of neuregulin isoforms containing an immunoglobulin-like domain are myasthenic [23], [24]. In *Dp1* mice, both Ig-Nrg1 and CRD-Nrg1 EGFβ-type isoforms are affected. Although myasthenia of skeletal muscle due to inactivation of Ig-NRG1 isoforms in mice has been previously reported, we cannot confirm the exact mutation where NRG1 isoforms are responsible for the abnormal phenotype caused by the reduction of M receptors in smooth muscles.

As a whole, the phenotype of affected heterozygous mice is milder than that of homozygous mice, and the mutation can be described as a semi-dominant mutation with respect to the expressivity of the mutant phenotype. However, the *Dp1* dilated pupil phenotype is inherited with very low penetrance in heterozygous mice and with complete penetrance in homozygous mice. Knowledge of this interesting inheritance pattern will be helpful in establishing additional mutant mice lines and models of human genetic disease and can be applied to other organisms.

Having a knockout with a severe phenotype (complete loss-of-function) is both advantageous and disadvantageous; it is an advantage because it provides reassurance that the gene of interest has an essential role, and it is a disadvantage because death or early developmental disruptions in the mutants preclude the analysis of later developmental events. The homozygous lethality of pan-NRG1, Ig-NRG1 and CRD-NRG1 KO mice hampers further knowledge of *Nrg1* function. *Dp1* is a partial loss-of-function mouse model and approximately 50% of *Dp1* homozygous mice are viable, which makes these mice a valuable resource for further clarifying the biological functions of *Nrg1*.

### Conclusion

We have identified a mutant mouse, *Dp1*, with a dilated pupil phenotype. The abnormal phenotype is caused by a base substitution flanking the exon encoding for the EGFβ domain, which affects the splicing of EGFβ-type *Nrg1* mRNA. Protein isoforms are expected, and the results show a decrease in higher affinity EGFβ-type Nrg1 isoforms. These are partially compensated for by increased expression of the lower affinity alpha forms or inactive proteins, suggesting that *Dp1* is a partial loss-of-function mouse model and that the mutation results in a hypomorphic allele. Further tests showed a reduction of M receptors in the iris constrictor muscles of *Dp1* mice, which is a major cause of the dilated pupil phenotype. The *Dp1* dilated pupil phenotype is inherited with very low penetrance in heterozygous mice and with complete penetrance in homozygous mice. This interesting inheritance pattern could be helpful in establishing more mutant mouse lines and models of human genetic diseases and can be applied to other organisms. *Dp1* mice are a valuable resource for further clarifying the biological functions of *Nrg1*.

## Materials and Methods

### Ethics statement

C57BL/6J (B6), C3He/J (C3H) and DBA/2J (D2) mice were obtained from the Shanghai Laboratory Animal Center (Shanghai, China). This study was conducted in strict accordance with the recommendations given in the Guide for the Care and Use of Laboratory Animals of the National Research Council. The animal care and use committee of Yangzhou University approved all experiments and procedures conducted on the animals (approval ID: SYXK (Su) 2007-0005).

### Preparation of DNA and RNA

Genomic DNA was isolated from mouse tail tips by proteinase K digestion, phenol chloroform extraction, and ethanol precipitation. Total RNA was extracted from the brains of *Dp1* and wild-type mice using TRIzol reagent (Invitrogen, Carlsbad, CA).

### Genetic mapping and mutation screening

As a first step for assigning a chromosomal location to the mutant, we crossed *Dp1* animals on the B6 genetic background to C3He/J (C3H) or DBA/2J (D2) mice. F1 offspring were then backcrossed to wild type (+/+) B6 animals to obtain BCB or BDB N2 mice. DNA from N2 offspring exhibiting dilated pupils was used to scan the genomic sequence. Due to a very low penetrance in this kind of mating strategy, after mapping the mutation to a chromosome, we tried to outcross *Dp1* animals on the B6 genetic background to C3H mice or D2 to obtain F1 mice and then crossbreed the F1 mice to obtain an F2. DNA of F2 offspring exhibiting dilated pupils was used to refine the mutant gene to a critical region. These genes in the region were then sequenced.

### Reverse transcription–polymerase chain reaction

The cDNA was synthesized using the RevertAid First Strand cDNA Synthesis Kit (Fermentas, EU) with Olig(dT)18 primers. RT-PCR for *CRD-Nrg1* was performed with the following primers: F1 5′-CCAAGTCAGGAACTCAGCCACAAA-3′ and R1 5′-CGCTATGTTCACCATGTTGTTTCGT-3′. RT-PCR for *Ig-Nrg1* was conducted with the primers F2 5′-CGTAGGAATAAACCACAAAACGTC-3′ and R2 5′-GAGCCGATCATGAAGCTTCTGCCG-3′. RT-PCR control reactions specific for the housekeeping gene *Gapdh* were preformed with the following primers: 5′-CTTTGGCATTGTGGAAGGG-3′ and 5′- CCTCTCTTGCTGCAGTGTC-3′.

### Genotyping of mice

The point mutation in *Dp1* abolished a *TaiI* restriction site present in the wild-type sequence. A 360-bp fragment encompassing the point mutation was amplified from genomic DNA using a forward primer, 5′-TCTGTCAGTGACACTACAGGAGCTC-3′, and reverse primer, 5′-GTAACTCAGAGCCGACTAGTCACA-3′. Digestion of the PCR product with *Tai* I was predicted to give DNA fragments of the following sizes: +/+ mice - three bands of 200 bp, 137 bp and 23 bp (the 360 bp PCR product of +/+ mice contains two *TaiI* restriction sites); heterozygotes - four bands of 223 bp, 200 bp, 137 bp and 23 bp; and *Dp1/Dp1* mice - two bands of 223 bp and 137 bp. A 4% gel electrophoresis revealed DNA bands with sizes consistent with this prediction.

### Immunohistochemistry

Eyeballs were fixed in a 4% paraformaldehyde solution in PBS, dehydrated, wax-embedded and sectioned at 6 µm. A rabbit polyclonal antibody against Chrm3 (Bioss, Beijing, China) was used to label Chrm3. Immunohistochemical analyses was performed using the Boster (Wuhang, China) reagents according to the manufacturer's instructions. The sections were visualized by incubation with 3-amino-9-ethylcarbazole (AEC) and were counterstained with Mayer's hematoxylin.

## References

[1] Justice MJ, Noveroske JK, Weber JS, Zheng B, Bradley A (1999). Mouse ENU mutagenesis.. Hum Mol Genet.

[2] Hrabé de Angelis MH, Flaswinkel H, Fuchs H, Rathkolb B, Soewarto D (2000). Genome-wide, large-scale production of mutant mice by ENU mutagenesis.. Nat Genet.

[3] Balling R (2001). ENU mutagenesis: analyzing gene function in mice.. Annu Rev Genomics Hum Genet.

[4] Nolan PM, Peters J, Strivens M, Rogers D, Hagan J (2000). A systematic, genome-wide, phenotype-driven mutagenesis programme for gene function studies in the mouse.. Nat Genet.

[5] Thaung C, West K, Clark BJ, McKie L, Morgan JE (2002). Novel ENU-induced eye mutations in the mouse: models for human eye disease.. Hum Mol Genet.

[6] Busfield SJ, Michnick DA, Chickering TW, Revett TL, Ma J (1997). Characterization of a neuregulin-related gene, Don-1, that is highly expressed in restricted regions of the cerebellum and hippocampus.. Mol Cell Biol.

[7] Carraway KL, Weber JL, Unger MJ, Ledesma J, Yu N (1997). Neuregulin-2, a new ligand of ErbB3/ErbB4-receptor tyrosine kinases.. Nature.

[8] Falls DL (2003). Neuregulins: functions, forms, and signaling strategies.. Exp Cell Res.

[9] Fischbach GD, Rosen KM (1997). ARIA: a neuromuscular junction neuregulin.. Annu Rev Neurosci.

[10] Harari D, Tzahar E, Romano J, Shelly M, Pierce JH (1999). Neuregulin-4: a novel growth factor that acts through the ErbB-4 receptor tyrosine kinase.. Oncogene.

[11] Zhang D, Sliwkowski MX, Mark M, Frantz G, Akita R (1997). Neuregulin-3 (NRG3): a novel neural tissue-enriched protein that binds and activates ErbB4.. Proc Natl Acad Sci U S A.

[12] Chen YJ, Johnson MA, Lieberman MD, Goodchild RE, Schobel S (2008). Type III neuregulin-1 is required for normal sensorimotor gating, memory-related behaviors, and corticostriatal circuit components.. J Neurosci.

[13] Harrison PJ, Law AJ (2006). Neuregulin 1 and schizophrenia: genetics, gene expression, and neurobiology.. Biol Psychiatry.

[14] Steinthorsdottir V, Stefansson H, Ghosh S, Birgisdottir B, Bjornsdottir S (2004). Multiple novel transcription initiation sites for NRG1.. Gene.

[15] Kramer R, Bucay N, Kane DJ, Martin LE, Tarpley JE (1996). Neuregulins with an Ig-like domain are essential for mouse myocardial and neuronal development.. Proc Natl Acad Sci USA.

[16] Meyer D, Birchmeier C (1995). Multiple essential functions of neuregulin in development.. Nature.

[17] Wolpowitz D, Mason TB, Dietrich P, Mendelsohn M, Talmage DA (2000). Cysteine-rich domain isoforms of the neuregulin-1 gene are required for maintenance of peripheral synapses.. Neuron.

[18] Matsui M, Motomura D, Karasawa H, Fujikawa T, Jiang J (2000). Multiple functional defects in peripheral autonomic organs in mice lacking muscarinic acetylcholine receptor gene for the M3 subtype.. Proc Natl Acad Sci USA.

[19] Matsui M, Motomura D, Fujikawa T, Jiang J, Takahashi S (2002). Mice lacking M2 and M3 muscarinic acetylcholine receptors are devoid of cholinergic smooth muscle contractions but still viable.. J Neurosci.

[20] Davis-Silberman N, Ashery-Padan R (2008). Iris development in vertebrates; genetic and molecular considerations.. Brain Res.

[21] Pilar G, Nuñez R, McLennan IS, Meriney SD (1987). Muscarinic and nicotinic synaptic activation of the developing chicken iris.. J Neurosci.

[22] Hill CE, Klemm M, Edwards FR, Hirst GD (1993). Sympathetic transmission to the dilator muscle of the rat iris.. J Auton Nerv Syst.

[23] Falls DL, Rosen KM, Corfas G, Lane WS, Fischbach GD (1993). ARIA, a protein that stimulates acetylcholine receptor synthesis, is a member of the neu ligand family.. Cell.

[24] Sandrock AW, Dryer SE, Rosen KM, Gozani SN, Kramer R (1997). Maintenance of acetylcholine receptor number by neuregulins at the neuromuscular junction in vivo.. Science.

